# DeepNetBim: deep learning model for predicting HLA-epitope interactions based on network analysis by harnessing binding and immunogenicity information

**DOI:** 10.1186/s12859-021-04155-y

**Published:** 2021-05-05

**Authors:** Xiaoyun Yang, Liyuan Zhao, Fang Wei, Jing Li

**Affiliations:** 1 Department of Bioinformatics and Biostatistics, School of Life Sciences and Biotechnology, Shanghai Jiao Tong University, Shanghai, China; 2 Sheng Yushou Center of Cell Biology and Immunology, Joint International Research Laboratory of Metabolic and Developmental Sciences, School of Life Sciences and Biotechnology, Shanghai Jiao Tong University, Shanghai, China

**Keywords:** T cell epitope prediction, Network analysis, Deep learning

## Abstract

**Background:**

Epitope prediction is a useful approach in cancer immunology and immunotherapy. Many computational methods, including machine learning and network analysis, have been developed quickly for such purposes. However, regarding clinical applications, the existing tools are insufficient because few of the predicted binding molecules are immunogenic. Hence, to develop more potent and effective vaccines, it is important to understand binding and immunogenic potential. Here, we observed that the interactive association constituted by human leukocyte antigen (HLA)-peptide pairs can be regarded as a network in which each HLA and peptide is taken as a node. We speculated whether this network could detect the essential interactive propensities embedded in HLA-peptide pairs. Thus, we developed a network-based deep learning method called DeepNetBim by harnessing binding and immunogenic information to predict HLA-peptide interactions.

**Results:**

Quantitative class I HLA-peptide binding data and qualitative immunogenic data (including data generated from T cell activation assays, major histocompatibility complex (MHC) binding assays and MHC ligand elution assays) were retrieved from the Immune Epitope Database database. The weighted HLA-peptide binding network and immunogenic network were integrated into a network-based deep learning algorithm constituted by a convolutional neural network and an attention mechanism. The results showed that the integration of network centrality metrics increased the power of both binding and immunogenicity predictions, while the new model significantly outperformed those that did not include network features and those with shuffled networks. Applied on benchmark and independent datasets, DeepNetBim achieved an AUC score of 93.74% in HLA-peptide binding prediction, outperforming 11 state-of-the-art relevant models. Furthermore, the performance enhancement of the combined model, which filtered out negative immunogenic predictions, was confirmed on neoantigen identification by an increase in both positive predictive value (PPV) and the proportion of neoantigen recognition.

**Conclusions:**

We developed a network-based deep learning method called DeepNetBim as a pan-specific epitope prediction tool. It extracted the attributes of the network as new features from HLA-peptide binding and immunogenic models. We observed that not only did DeepNetBim binding model outperform other updated methods but the combination of our two models showed better performance. This indicates further applications in clinical practice.

**Supplementary Information:**

The online version contains supplementary material available at 10.1186/s12859-021-04155-y.

## Background

Accurate identification of peptide presentation to major histocompatibility complex (MHC) molecules is of great importance for exploring the mechanism of immune recognition [1]. The human leukocyte antigen (HLA) gene complex encodes MHC proteins in humans. Two main classes of HLA molecules are important in the immunological context. Class I molecules present epitopes to CD8^+^ T cells, while class II molecules present epitopes to CD4^+^ T cells. The verification methods for assessing immunogenicity include both MS-based MHC-I binding peptide detection and immunogenicity verification by specific T-cell response assays [2, 3]. Over the past 30 years, many studies have been devoted to predicting T cell recognition of MHC class I epitopes for immunological reactions. For this purpose, two types of computational methods have been developed: allele-specific and pan-specific methods. The former trains one model for every MHC-I allele [4–7], while the latter considers both peptides and MHCs and pools all different alleles together to train one general model [8–12]. Most accurate methods retrieve data from The Immune Epitope Database (IEDB) [13], where over 73% of the binding data are 9-mer peptides [12].

Many attempts have been made to improve prediction performance. Recently, an ever-increasing amount of mass spectrometry (MS)-based HLA peptidome data has become available. Therefore, there is growing interest in applying these data to peptide-HLA interaction studies [14]. Some methods also consider proteasomal cleavage and transporter-associated antigen processing (TAP)-mediated peptide transport [15–18]. In addition, with the rapid development of deep learning methods, shallow and high-order artificial neural networks have been proposed by various research teams, including NetMHC 4.0 [4], HLA-CNN [7], ConvMHC [8], NetMHCPan 4.0 [10], MHCflurry 1.2.0 [19], MHCnuggets [20] and NetMHCstabpan 1.0 [21].

It has been reported that high affinity MHC-epitope interactions tend to be associated with higher immune responsiveness [22]. However, even though MHC binding is necessary for recognition by T cells, it is in itself not sufficient to define immunogenicity. According to a previous report [23], a CD8^+^ T cell immunogenic peptide is able to form a complex with class I MHC molecules and trigger the activation and proliferation of T cells. Nevertheless, there is a lack of effective strategies available to predict immunogenicity [24]. The existing binding prediction tools are insufficient for neoantigen prediction in clinical applications considering that recognition is also influenced by several other factors, such as the abundance of proteins, immunodominance, antigen processing and the presence of a suitable T-cell repertoire [25–27]. Hence, top-ranking candidates predicted by binding affinity for alleles are usually falsely reported as neoantigens [28]. Thus, the demand to understand binding and immunogenic potential should be met eagerly for more potent and peptide-based vaccines.

Here, we observed that the interactive association constituted by HLA-peptide pairs can be regarded as a network in which each HLA and peptide is taken as a node. We believe that in this network, the features of nodes or edges are important and informative for detecting essential HLA-peptide interactive potentials. Thus, in this study, we developed a new network-based deep learning model called DeepNetBim (a deep learning model based on network analysis by harnessing binding and immunogenicity information) for accurate HLA class I pan-specific epitope presentation prediction. To overcome the previously explained deficiencies, we applied the weighted HLA-peptide binding network and immunogenic network into a network-based deep learning algorithm by combining binding and immunogenic models to predict HLA-peptide interactions efficiently.

Extensive tests on benchmark and independent datasets demonstrated that DeepNetBim binding model can significantly outperform other well-known binding prediction tools. Furthermore, the performance enhancement of the combined model, which filtered out negative immunogenic predictions, was confirmed on neoantigen identification by increases in both the positive predictive value (PPV) and the proportion of neoantigen recognition. Overall, DeepNetBim provides a powerful and useful tool for T cell epitope recognition and further immunotherapy practice.

## Results

**Fig. 1:**
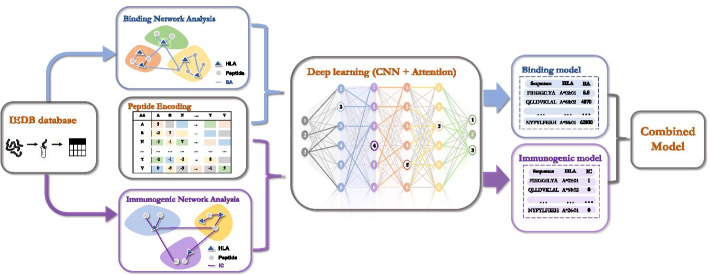
Workflow diagram of DeepNetBim framework. First, the binding and immunogenic data were retrieved from the IEDB database. Then, the weighted HLA-peptide binding network (coloured in blue) and immunogenic network (coloured in purple) were constructed separately to acquire quantified network features. Next, the encoded peptides and the obtained network features were fed into an attention-based deep learning process. Finally, the predicted binding affinity and binary immunogenic category of the above two independent models were combined to make the final prediction. *BA* binding affinity, *IC* immunogenic category

In this study, a new network-based deep learning model called DeepNetBim was proposed (in the following paragraphs, we use ‘HLA’ to refer to ‘HLA class I’ for convenience). The weighted HLA-peptide binding network and immunogenic network were applied to a network-based deep learning algorithm constituted by a convolutional neural network (CNN) and an attention mechanism (Fig. 1). First, the quantitative class I HLA-peptide binding data and qualitative immunogenic data were retrieved from the IEDB database. Then, the weighted HLA-peptide binding network and immunogenic network were constructed separately to acquire quantified individual network features. Next, the encoded peptides and the obtained network features were fed into an attention-based deep learning process. Finally, the predicted binding affinity and binary immunogenic categories of the abovementioned independent models were combined to make the final prediction (“Methods”).

### Evaluation of network metrics by stepwise regressions

In our model for predicting binding affinity and immunogenic category, we explored four traditional centrality metrics (degree, closeness, betweenness and eigenvector) for each HLA and peptide. To investigate the roles that the extracted network metrics may play in the binding or immunogenic propensities of HLA-peptide pairs, we applied two multiple linear models: a linear regression model and a logistic regression model for binding and immunogenic data separately. In these two models, the eight network attributes ($HL{A}_{degree}$, $HL{A}_{closeness}$, $HL{A}_{betweeness}$, $HL{A}_{eigenvector}$, $PE{P}_{degree}$, $PE{P}_{closeness}$, $PE{P}_{betweeness}$, $PE{P}_{eigenvetor}$) were taken as independent variables, while the transformed binding affinity (BA) was taken as the response variable in the linear regression model (Eq. 1), and immunogenicity category (IC) was taken as the binary response variable in the logistic regression model (Eq. 2) as following:

1 $$BA= {\boldsymbol{\alpha }}_{{\varvec{b}}}+\boldsymbol{ }{{\varvec{\beta}}}_{{\varvec{H}}{\varvec{L}}{{\varvec{A}}}_{{\varvec{d}}{\varvec{e}}{\varvec{g}}{\varvec{r}}{\varvec{e}}{\varvec{e}}}}.\boldsymbol{ }{{\varvec{x}}}_{{\varvec{H}}{\varvec{L}}{{\varvec{A}}}_{{\varvec{d}}{\varvec{e}}{\varvec{g}}{\varvec{r}}{\varvec{e}}{\varvec{e}}}}+{{\varvec{\beta}}}_{{\varvec{H}}{\varvec{L}}{{\varvec{A}}}_{{\varvec{c}}{\varvec{l}}{\varvec{o}}{\varvec{s}}{\varvec{e}}{\varvec{n}}{\varvec{e}}{\varvec{s}}{\varvec{s}}\boldsymbol{ }}}\bullet {{\varvec{x}}}_{{\varvec{H}}{\varvec{L}}{{\varvec{A}}}_{{\varvec{c}}{\varvec{l}}{\varvec{o}}{\varvec{s}}{\varvec{e}}{\varvec{n}}{\varvec{e}}{\varvec{s}}{\varvec{s}}}}+\boldsymbol{ }{{\varvec{\beta}}}_{{\varvec{H}}{\varvec{L}}{{\varvec{A}}}_{{\varvec{b}}{\varvec{e}}{\varvec{t}}{\varvec{w}}{\varvec{e}}{\varvec{e}}{\varvec{n}}{\varvec{e}}{\varvec{s}}{\varvec{s}}}}\bullet {{\varvec{x}}}_{{\varvec{H}}{\varvec{L}}{{\varvec{A}}}_{{\varvec{b}}{\varvec{e}}{\varvec{t}}{\varvec{w}}{\varvec{e}}{\varvec{e}}{\varvec{n}}{\varvec{e}}{\varvec{s}}{\varvec{s}}}}+{{\varvec{\beta}}}_{{\varvec{H}}{\varvec{L}}{{\varvec{A}}}_{{\varvec{e}}{\varvec{i}}{\varvec{g}}{\varvec{e}}{\varvec{n}}{\varvec{v}}{\varvec{e}}{\varvec{c}}{\varvec{t}}{\varvec{o}}{\varvec{r}}\boldsymbol{ }\boldsymbol{ }}}\bullet {{\varvec{x}}}_{{\varvec{H}}{\varvec{L}}{{\varvec{A}}}_{{\varvec{e}}{\varvec{i}}{\varvec{g}}{\varvec{e}}{\varvec{n}}{\varvec{v}}{\varvec{e}}{\varvec{c}}{\varvec{t}}{\varvec{o}}{\varvec{r}}\boldsymbol{ }\boldsymbol{ }}}+{{\varvec{\beta}}}_{{\varvec{P}}{\varvec{E}}{{\varvec{P}}}_{{\varvec{d}}{\varvec{e}}{\varvec{g}}{\varvec{r}}{\varvec{e}}{\varvec{e}}}}\bullet {{\varvec{x}}}_{{\varvec{P}}{\varvec{E}}{{\varvec{P}}}_{{\varvec{d}}{\varvec{e}}{\varvec{g}}{\varvec{r}}{\varvec{e}}{\varvec{e}}}}+{{\varvec{\beta}}}_{{\varvec{P}}{\varvec{E}}{{\varvec{P}}}_{{\varvec{c}}{\varvec{l}}{\varvec{o}}{\varvec{s}}{\varvec{e}}{\varvec{n}}{\varvec{e}}{\varvec{s}}{\varvec{s}}}}\bullet {{\varvec{x}}}_{{\varvec{P}}{\varvec{E}}{{\varvec{P}}}_{{\varvec{c}}{\varvec{l}}{\varvec{o}}{\varvec{s}}{\varvec{e}}{\varvec{n}}{\varvec{e}}{\varvec{s}}{\varvec{s}}}}+{{\varvec{\beta}}}_{{\varvec{P}}{\varvec{E}}{{\varvec{P}}}_{{\varvec{b}}{\varvec{e}}{\varvec{t}}{\varvec{w}}{\varvec{e}}{\varvec{e}}{\varvec{n}}{\varvec{e}}{\varvec{s}}{\varvec{s}}}}\bullet {{\varvec{x}}}_{{\varvec{P}}{\varvec{E}}{{\varvec{P}}}_{{\varvec{b}}{\varvec{e}}{\varvec{t}}{\varvec{w}}{\varvec{e}}{\varvec{e}}{\varvec{n}}{\varvec{e}}{\varvec{s}}{\varvec{s}}}}+{{\varvec{\beta}}}_{{\varvec{P}}{\varvec{E}}{{\varvec{P}}}_{{\varvec{e}}{\varvec{i}}{\varvec{g}}{\varvec{e}}{\varvec{n}}{\varvec{v}}{\varvec{e}}{\varvec{t}}{\varvec{o}}{\varvec{r}}\boldsymbol{ }}}\bullet {{\varvec{x}}}_{{\varvec{P}}{\varvec{E}}{{\varvec{P}}}_{{\varvec{e}}{\varvec{i}}{\varvec{g}}{\varvec{e}}{\varvec{n}}{\varvec{v}}{\varvec{e}}{\varvec{t}}{\varvec{o}}{\varvec{r}}}}$$

2 $$\mathrm{log}\left(\frac{\theta }{1-\theta }\right)= {\boldsymbol{\alpha }}_{{\varvec{i}}}+\boldsymbol{ }{{\varvec{\gamma}}}_{{\varvec{H}}{\varvec{L}}{{\varvec{A}}}_{{\varvec{d}}{\varvec{e}}{\varvec{g}}{\varvec{r}}{\varvec{e}}{\varvec{e}}}}.\boldsymbol{ }{{\varvec{x}}}_{{\varvec{H}}{\varvec{L}}{{\varvec{A}}}_{{\varvec{d}}{\varvec{e}}{\varvec{g}}{\varvec{r}}{\varvec{e}}{\varvec{e}}}}+{{\varvec{\gamma}}}_{{\varvec{H}}{\varvec{L}}{{\varvec{A}}}_{{\varvec{c}}{\varvec{l}}{\varvec{o}}{\varvec{s}}{\varvec{e}}{\varvec{n}}{\varvec{e}}{\varvec{s}}{\varvec{s}}\boldsymbol{ }}}\bullet {{\varvec{x}}}_{{\varvec{H}}{\varvec{L}}{{\varvec{A}}}_{{\varvec{c}}{\varvec{l}}{\varvec{o}}{\varvec{s}}{\varvec{e}}{\varvec{n}}{\varvec{e}}{\varvec{s}}{\varvec{s}}}}+\boldsymbol{ }{{\varvec{\gamma}}}_{{\varvec{H}}{\varvec{L}}{{\varvec{A}}}_{{\varvec{b}}{\varvec{e}}{\varvec{t}}{\varvec{w}}{\varvec{e}}{\varvec{e}}{\varvec{n}}{\varvec{e}}{\varvec{s}}{\varvec{s}}}}\bullet {{\varvec{x}}}_{{\varvec{H}}{\varvec{L}}{{\varvec{A}}}_{{\varvec{b}}{\varvec{e}}{\varvec{t}}{\varvec{w}}{\varvec{e}}{\varvec{e}}{\varvec{n}}{\varvec{e}}{\varvec{s}}{\varvec{s}}}}+{{\varvec{\gamma}}}_{{\varvec{H}}{\varvec{L}}{{\varvec{A}}}_{{\varvec{e}}{\varvec{i}}{\varvec{g}}{\varvec{e}}{\varvec{n}}{\varvec{v}}{\varvec{e}}{\varvec{c}}{\varvec{t}}{\varvec{o}}{\varvec{r}}\boldsymbol{ }\boldsymbol{ }}}\bullet {{\varvec{x}}}_{{\varvec{H}}{\varvec{L}}{{\varvec{A}}}_{{\varvec{e}}{\varvec{i}}{\varvec{g}}{\varvec{e}}{\varvec{n}}{\varvec{v}}{\varvec{e}}{\varvec{c}}{\varvec{t}}{\varvec{o}}{\varvec{r}}\boldsymbol{ }\boldsymbol{ }}}+{{\varvec{\gamma}}}_{{\varvec{P}}{\varvec{E}}{{\varvec{P}}}_{{\varvec{d}}{\varvec{e}}{\varvec{g}}{\varvec{r}}{\varvec{e}}{\varvec{e}}}}\bullet {{\varvec{x}}}_{{\varvec{P}}{\varvec{E}}{{\varvec{P}}}_{{\varvec{d}}{\varvec{e}}{\varvec{g}}{\varvec{r}}{\varvec{e}}{\varvec{e}}}}+{{\varvec{\gamma}}}_{{\varvec{P}}{\varvec{E}}{{\varvec{P}}}_{{\varvec{c}}{\varvec{l}}{\varvec{o}}{\varvec{s}}{\varvec{e}}{\varvec{n}}{\varvec{e}}{\varvec{s}}{\varvec{s}}}}\bullet {{\varvec{x}}}_{{\varvec{P}}{\varvec{E}}{{\varvec{P}}}_{{\varvec{c}}{\varvec{l}}{\varvec{o}}{\varvec{s}}{\varvec{e}}{\varvec{n}}{\varvec{e}}{\varvec{s}}{\varvec{s}}}}+{{\varvec{\gamma}}}_{{\varvec{P}}{\varvec{E}}{{\varvec{P}}}_{{\varvec{b}}{\varvec{e}}{\varvec{t}}{\varvec{w}}{\varvec{e}}{\varvec{e}}{\varvec{n}}{\varvec{e}}{\varvec{s}}{\varvec{s}}}}\bullet {{\varvec{x}}}_{{\varvec{P}}{\varvec{E}}{{\varvec{P}}}_{{\varvec{b}}{\varvec{e}}{\varvec{t}}{\varvec{w}}{\varvec{e}}{\varvec{e}}{\varvec{n}}{\varvec{e}}{\varvec{s}}{\varvec{s}}}}+{{\varvec{\gamma}}}_{{\varvec{P}}{\varvec{E}}{{\varvec{P}}}_{{\varvec{e}}{\varvec{i}}{\varvec{g}}{\varvec{e}}{\varvec{n}}{\varvec{v}}{\varvec{e}}{\varvec{t}}{\varvec{o}}{\varvec{r}}\boldsymbol{ }}}\bullet {{\varvec{x}}}_{{\varvec{P}}{\varvec{E}}{{\varvec{P}}}_{{\varvec{e}}{\varvec{i}}{\varvec{g}}{\varvec{e}}{\varvec{n}}{\varvec{v}}{\varvec{e}}{\varvec{t}}{\varvec{o}}{\varvec{r}}\boldsymbol{ }}}\boldsymbol{ }$$

where $\theta =P\left(IC=1|x\right)$, and ${\alpha }_{b}$ and ${\alpha }_{i}$ are intercepts of the two models. $\beta$ and $\gamma$ represent the attribute coefficients in the binding and immunogenic regression models, respectively. During each model’s construction, stepwise regression was applied. The stepwise procedure helps to extract the primary factors that may affect binding or immunogenicity and remove the variables that were not statistically significant. In addition, it avoids selecting a variable that is highly correlated to selected variables. The fitted coefficients are shown in Table 1. All of those variables were selected through stepwise selections in both models with $p<2\times {10}^{-16}$, which confirms the necessities of all these variables. Furthermore, the increase in positive coefficients (such as ${{\varvec{\beta}}}_{{\varvec{H}}{\varvec{L}}{{\varvec{A}}}_{{\varvec{c}}{\varvec{l}}{\varvec{o}}{\varvec{s}}{\varvec{e}}{\varvec{n}}{\varvec{e}}{\varvec{s}}{\varvec{s}}}}$**, **${{\varvec{\beta}}}_{{\varvec{H}}{{\varvec{L}}{\varvec{A}}}_{{\varvec{b}}{\varvec{e}}{\varvec{t}}{\varvec{w}}{\varvec{e}}{\varvec{e}}{\varvec{n}}{\varvec{e}}{\varvec{s}}{\varvec{s}}}}$**, **${{\varvec{\beta}}}_{{\varvec{H}}{\varvec{L}}{{\varvec{A}}}_{{\varvec{e}}{\varvec{i}}{\varvec{g}}{\varvec{e}}{\varvec{n}}{\varvec{v}}{\varvec{e}}{\varvec{c}}{\varvec{t}}{\varvec{o}}{\varvec{r}}}}$**, **${{\varvec{\beta}}}_{{{\varvec{P}}{\varvec{E}}{\varvec{P}}}_{{\varvec{e}}{\varvec{i}}{\varvec{g}}{\varvec{e}}{\varvec{n}}{\varvec{v}}{\varvec{e}}{\varvec{c}}{\varvec{t}}{\varvec{o}}{\varvec{r}}}}$**, **${{\varvec{\gamma}}}_{{\varvec{H}}{\varvec{L}}{{\varvec{A}}}_{{\varvec{d}}{\varvec{e}}{\varvec{g}}{\varvec{r}}{\varvec{e}}{\varvec{e}}}}$**, **${{\varvec{\gamma}}}_{{\varvec{P}}{\varvec{E}}{{\varvec{P}}}_{{\varvec{c}}{\varvec{l}}{\varvec{o}}{\varvec{s}}{\varvec{e}}{\varvec{n}}{\varvec{e}}{\varvec{s}}{\varvec{s}}}}$**,** ${{\varvec{\gamma}}}_{{\varvec{P}}{\varvec{E}}{{\varvec{P}}}_{{\varvec{b}}{\varvec{e}}{\varvec{t}}{\varvec{w}}{\varvec{e}}{\varvec{e}}{\varvec{n}}{\varvec{e}}{\varvec{s}}{\varvec{s}}}}$) was significantly associated with increased binding and immunogenic propensities of HLA-peptide pairs and vice versa.

**Table 1:** Coefficients of network metrics in two multiple regression models

Binding (linear regression)	HLA attributes					
	Variable	${\alpha }_{b}$	${\beta }_{HL{A}_{degree}}$	${\beta }_{HL{A}_{closeness}}$	${\beta }_{HL{A}_{betweeness}}$	${\beta }_{HL{A}_{eigenvector}}$
	coefficient	0.281	− 0.048	0.095	0.02	0.003
	Peptide attributes					
	Variable		${\beta }_{PE{P}_{degree}}$	${\beta }_{PE{P}_{closeness}}$	${\beta }_{PE{P}_{betweeness}}$	${\beta }_{{PEP}_{eigenvector}}$
	Coefficient		− 0.044	− 0.173	− 0.003	0.05
Immunogenic (logistic regression)	HLA attributes					
	Variable	${\alpha }_{i}$	${\gamma }_{HL{A}_{degree}}$	${\gamma }_{HL{A}_{closeness}}$	${\gamma }_{H{LA}_{betweeness}}$	${\gamma }_{HL{A}_{eigenvector}}$
	Coefficient	1.914	5.241	− 1.878	− 4.083	− 2.242
	Peptide attributes					
	Variable		${\gamma }_{PE{P}_{degree}}$	${\gamma }_{PE{P}_{closeness}}$	${\gamma }_{PE{P}_{betweeness}}$	${\gamma }_{{PEP}_{eigenvector}}$
	Coefficient		− 0.429	3.791	0.092	− 1.022

The correlation between the metrics is shown in Additional file 1: Fig. S1. The correlation among network metrics was higher amongst HLA attributes and amongst peptide attributes than between attributes of these two distinct groups. Furthermore, the density distribution of network metrics of HLA and peptides in positive (binder/immunogenic) and negative (nonbinder/nonimmunogenic) datasets of the two models are visualized in Additional file 1: Fig. S2. Large differences in the density distribution of positive and negative datasets implied that there might be large associations among network metrics and different binding and immunogenic categories (*p* value < 0.001 by Kolmogorov–Smirnov test). In addition, we applied the chi-squared test on network metrics and their corresponding categories (see Additional file 2: Table S1). A statistically significant result (*p* value < 0.001) indicated that the network centrality metrics might reveal some intrinsic interactive potentials embedded in HLA-peptide pairs. All of these results illustrated that the network metrics were crucial components for HLA-peptide interaction predictions.

### Improved performance by DeepNetBim

The binding and immunogenic models both achieved good performance on each test set with 0.015 mean absolute error on the binding model and 94.7% accuracy on the immunogenic model on average based on fivefold cross-validation. In addition, to better investigate how the performance improved by integrating network metrics in DeepNetBim, we compared the DeepNetBim model with its submodel and the transformed models we mentioned in the “Methods” section.

**Fig. 2:**
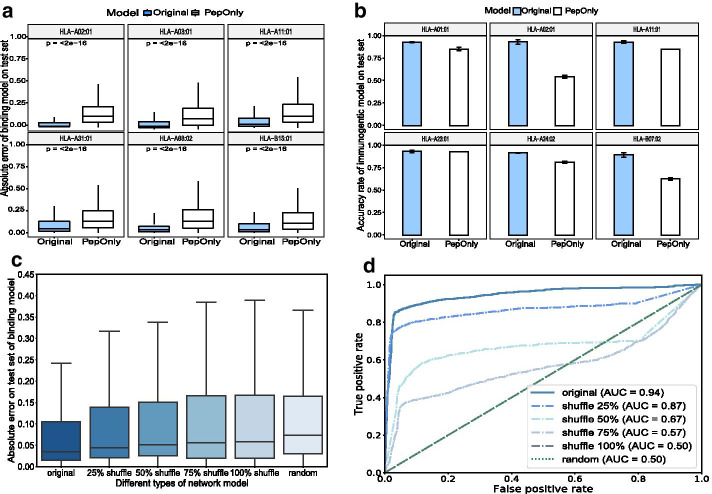
Improved performance of integrating network metrics in DeepNetBim. **a** Absolute error for binding affinity prediction and **b** accuracy rate for immunogenic prediction of the original model (coloured in blue) and the PepOnly model (coloured in white) for the 6 most frequent alleles in the test dataset. **c** Absolute error for binding affinity prediction and **d** ROC curve for immunogenic prediction of the original network, shuffle networks (shuffled network edge weights) and random network (reassigned network weights by uniform distribution) in the test dataset

We first compared the full model (original model) of DeepNetBim with its submodel, namely, the PepOnly model, which solely utilized encoded peptide sequence information without integrating network metric features. As expected, the original DeepNetBim achieved a significant improvement over PepOnly, both in the binding model and the immunogenic model with lower absolute error and higher accuracy rate, respectively. Figure 2a, b show performance comparisons of the 6 most frequently appearing alleles in the binding model and in the immunogenic model. Furthermore, we constructed several submodels, each missing one of the network metrics, and we compared them to DeepNetBim during the deep learning training. In each case, the original model achieved better performance. This confirmed the contribution of each of the network metrics (shown in Additional file 1: Fig. S3).

In the network construction section, we proposed two other transformed models: a shuffle model and a random model (see “Methods” section). After this transformation, the features of the newly obtained network metrics were fed along with the encoded peptides into the deep learning neural network to obtain the final result. Through comparison, we found that both the shuffle networks and the random networks performed poorly in terms of absolute error (binding model) and the area under the ROC curve (AUC) (immunogenic model) (Fig. 2c, d). Although the overall network metric values in the shuffle network model remained the same as in the original model, they were still remarkably outperformed by DeepNetBim. In addition, we observed that the higher shuffle levels translated into worse performance. The random network behaved similarly to the 100% shuffle model to some extent. Such a comparison indicated the necessity to assign the network edge weights correctly and effectively. All of these results demonstrated that integrating network metrics in DeepNetBim can effectively boost the prediction performance for not only HLA-peptide binding but also peptide immunogenicity.

### Evaluation of DeepNetBim binding model on the independent datasets

**Fig. 3:**
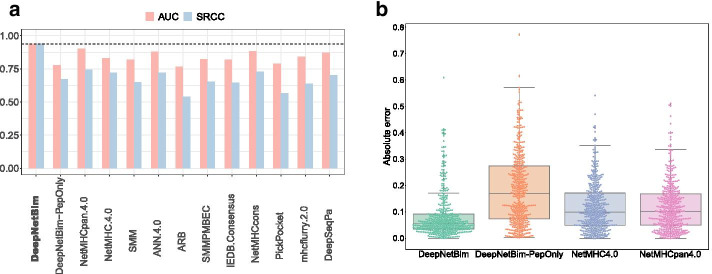
Performance comparison on the benchmark dataset and the external dataset. **a** Performance of the binding model compared with PepOnly and other tools on the latest IEDB benchmark dataset in terms of AUC and SRCC. **b** Performance compared with PepOnly, NetMHC4.0 and NetMHCpan4.0 on external data in terms of absolute error value

The IEDB has an automatic server benchmark page that evaluates different predictive methods for HLA-peptide binding pairs. We used the latest dataset whose measurement type was IC50 (http://tools.iedb.org/auto_bench/mhci/weekly/single/2019-03-15). Other updated benchmark datasets only contain binary data; therefore, they are not suitable for binding affinity prediction comparison. The dataset contains 434 peptides, 368 of which were positive. These data were isolated from the training section of the network construction phase. Two performance metrics, Spearman’s rank correlation coefficient (SRCC) and AUC, were retrieved between the predicted binding affinity and experimentally validated values. The DeepNetBim binding model achieved a comparable performance and 11 other well-acknowledged prediction tools with 0.273 improvement in terms of SRCC and 0.1 improvement in terms of AUC on average. Remarkably, two widely used tools, NetMHC4.0 [4] and NetMHCpan 4.0 [10], achieved SRCC values of 0.737 on average, while the DeepNetBim binding model achieved 0.9374 (Fig. 3a). On the benchmark dataset, DeepNetBim also significantly outperformed the PepOnly model. It confirmed the necessity of network metrics.

Then, we investigated the performance of the DeepNetBim binding model on independent data from an external study [29] compared to the PepOnly model and the two most frequently used machine learning algorithms: NetMHC4.0 [4] and NetMHCpan4.0 [10]. The dataset contained 434 nonduplicate 9-mer HLA-A*0201-restricted CD8 + T cell epitopes from the herpes simplex virus type 1 (HSV-1) genome. Among all 434 HLA-peptide pairs, the DeepNetBim binding model made more accurate predictions on 288 of them with lower absolute error compared with NetMHC4.0 and 293 of them compared with NetMHCpan4.0 (Fig. 3b). Between NetMHCpan4.0 and DeepNetBim, we observed differences in prediction in 160 pairs greater than 0.1, of which 77.5% (124) were predicted more accurately by our model. Similar results were obtained from DeepNetBim after we compared it with NetMHC4.0 (157 pairs showed a difference greater than 0.1, of which 79.6% were better predicted by DeepNetBim). In general, the DeepNetBim binding model showed significantly better performance (*p* value < 0.001 by *t*-test) both with NetMHC4.0 and with NetMHCpan4.0. The results demonstrated that DeepNetBim possessed relatively high generalizability for binding prediction.

### The combined model showed enhanced performance

**Fig. 4:**
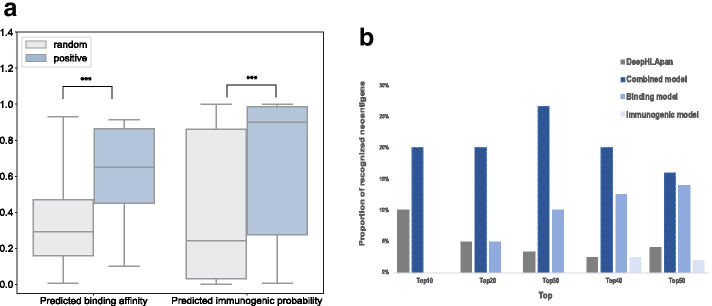
Performance on the separate models and the combined model. **a** Boxplot of positive and random peptides in the binding and the immunogenic model with *p* value < 0.001 by one-sided Mann–Whitney *U* test. **b** The proportion of recognized neoantigens in the top-ranked 10, 20, 30, 40 and 50 neoantigen candidates

In the Methods section, we proposed the combined model, which regarded the use of the immunogenic model as a filter. Here, to investigate how effective the combined model is, we applied it to the independent dataset. The independent data used to evaluate the combined model were obtained from Koşaloğlu-Yalçın, which contains 41 9-mer neoantigens (positive) with 1600 random 9-mer peptides (random) collected from the publicly available The Cancer Genome Atlas (TCGA) data portal [30]. Figure 4a depicts the predictive performance of our two separate models on this independent dataset. Both binding and immunogenic models significantly distinguished positive samples from random samples (*p* value < 0.001 by one-sided Mann–Whitney *U* test).

Predicted peptides were categorized into predicted binders/immunogenic (positives, P) or predicted nonbinders/nonimmunogenic (negatives, N) for each model. Based on experimental validation, these predictions were then split into four categories: true positives (TP), true negatives (TN), false positives (FP) and false negatives (FN). Four commonly used performance metrics for evaluating algorithms were applied: accuracy, sensitivity, specificity and positive predictive value (PPV). These four are defined as follows: accuracy = (TP + TN)/(P + N), sensitivity = TP/(TP + FN), specificity = TN/(TN + FP), PPV = TP/(TP + FP).

Table 2 shows the model performance measured by the four abovementioned metrics. According to [18], the rarer the event to be predicted, the harder it is to achieve high PPV prediction. Through model combination, we were able to improve the PPV from 6.1% of the binding model and 3.8% of the immunogenic model to 8.1% by using the combined model.

**Table 2:** Performance on combined model

	Binding model	Immunogenic model	Combined model
Accuracy	0.695	0.627	0.676
Sensitivity	0.780	0.585	0.708
Specificity	0.694	0.628	0.676
PPV	0.061	0.038	0.081

The percentile ranks of binding and immunogenic models were derived across all neoantigen candidates. We compared the predictive methods by calculating the proportion of identified neoantigens capable of eliciting CD8 + T cell responses in the top-ranked 10, 20, 30, 40 and 50. In 2019, Wu et al. [31] reused this dataset and obtained comparable performance. Additionally, in their study, they provided predicted immunogenic scores for neoantigen identification according to their method named DeepHLApan. Compared with their study [31], the final result indicates that our combined model exhibits a better performance than that achieved by DeepHLApan (Fig. 4b). In addition, it is noted that the separate models recognized no neoantigens in the top 10 candidates, while the combined model improved recognition to 20%.

## Discussion

With the development of cancer immunotherapy, it is essential to predict which of the peptides presented on the cell surface could be targeted for the T-cell response. In this study, we developed a network-based deep learning method by harnessing binding and immunogenicity information to obtain improved HLA-epitope interaction prediction. When investigating network structures, we utilized centrality metrics to quantify the “importance” or “influence” of nodes and edges. Network metrics have been used in many fields, such as contagion phenomena and social communities [32–34]. The stepwise regression of the extracted attributes of the network effectively indicates the binding or immunogenic propensities in the HLA-peptide interaction process, which confirms the significance of network exploration. The great improvement of DeepNetBim by correctly incorporating network centrality metrics validated the contribution of newly added network features. In addition, DeepNetBim achieved better performance compared with other well-known tools both on the IEDB benchmark dataset and the external dataset on binding affinity prediction. The fact that our original model outperformed several submodels may also explain why it was better than other published tools that only make use of deep learning methods. Furthermore, when we calculated both the PPV and the proportion of identified neoantigens, we confirmed an increased performance by combining the HLA binding and immunogenic models.

However, the DeepNetBim algorithm is only applicable to 9-mer peptides considering that the majority of MHC class I peptide-binding data are 9-mers in IEDBs [12]. Insufficient training data on the remaining lengths led us to unsatisfactory performances after several attempts. Nevertheless, the outperformance of DeepNetBim compared with other state-of-the-art tools on 9-mer peptides indicates that padding or deletion strategies may work on 8-mer, 10-mer or 11-mer peptides, which is worth trying in future endeavours. On the other hand, other types of influences that have been reported previously, such as N- or C-terminal extensions [35], are not included in our study.

In addition, some potentially immunogenic peptides may not have been recognized owing to the limited response rate, inefficient T cell priming and heterogeneity in the tumour fractions [36]. Thus, the risk introduced by potential false negative immunogenic peptides should be noted. To balance the sensitivity and specificity of the predictive algorithm, an adjustable weighting factor could be applied to integrate the binding and the immunogenic model. Furthermore, as we described in the Methods section, the IEDB database captured the results of the MHC assay and T cell activation assay. To date, limited by the training size of the immunogenic data, two types of assays were pooled together for the following deep learning process. In the future, if the amount of new immunogenic resources increases substantially, we can build specific predictive models for the different types of epitope assays.

Although this novel method has limitations, it is the first study to integrate network features with a deep learning algorithm to better interpret and understand HLA-peptide interactions. Even though we have demonstrated how effectively DeepNetBim can be applied to predict HLA-peptide interactions, we speculate that the well-designed ensemble classifier of DeepNetBim and previous work may provide a sustainable framework and increase the accuracy in further research. We anticipate that network-based deep-learning approaches such as the one presented in this work will play increasingly important roles in future studies.

## Conclusions

We developed a network-based deep learning method called DeepNetBim as a pan-specific epitope prediction tool. It extracted the attributes of the network as new features from HLA-peptide binding and immunogenic models. We observed that not only did DeepNetBim binding model outperform other updated methods but the combination of our two models showed better performance. This indicates further applications in clinical practice.

## Methods

### Binding and immunogenic data

The binding affinity dataset was collected from the IEDB (http://www.iedb.org/) [13]. To generate a pan-specific binding model, we kept only 9-mer peptides, as they collectively represent 73% of the total peptide binding data [12]. The resulting size is suitable for the training step in the following deep learning process. Thus, 104 HLA alleles were retrieved, with each allele having more than 5 peptide binding entries, and 88,913 nonredundant HLA-peptide pairs were obtained after duplicated data were removed.

All peptide binding affinities were measured by half-maximal inhibitory concentration (IC50) values. For this study, the binding affinity was log-transformed to fall in the range between 0 and 1 by using the relation 1− log(*x*)/log (50,000), where *x* is the measured binding affinity [37]. Based on strong biological support [38], we used an IC50 threshold of 500 nM (binder ≤ 500 nM) for binary classification. Thus, with this kind of log-transformation, a measured affinity less than 500 nM was assigned an output value above 0.426.

The immunogenic data were retrieved from IEDB. The immune epitope assay data we collected included data from T cell activation assays, MHC binding assays and MHC ligand elution assays. The immunogenic data were generated based on infectious pathogens, diseases, allergens and autoantigens. These binary data were used to create an immunogenic model that provided immunogenic or nonimmunogenic results. A total of 119 HLA alleles were retrieved, with each allele having more than 5 peptide entries. A total of 24,193 nonredundant HLA-peptide pairs were obtained after duplicated data were removed (only considering 9-mer peptides).

### Network construction of binding and immunogenic data

#### Original network construction

In the original network construction of DeepNetBim, the interactive association constituted by HLA-peptide pairs could be regarded as a network in which each HLA and peptide was taken as a node. The binding network and the immunogenic network were constructed by applying identical methods separately. In the binding model, the network weight was assigned by the transformed affinity (range from 0 to 1), while in the immunogenic model, it was assigned by the immunogenic binary category (0/1) instead. Additional file 1: Fig. S4 illustrates the network construction process. The weighted HLA-peptide network was established by using the igraph package (version 1.2.4.2) [39] in R (version 3.6.1). However, considering that only positive values are allowed in the igraph package, in the immunogenic model, the weight of the nonimmunogenic peptides with a 0 value was substituted by a small positive value close to 0 (which is ${10}^{-10}$ in our work, as an approximation method), while the weight of immunogenic peptides was set to 1 [40].

In DeepNetBim, the binding network and the immunogenic network were constructed separately but processed in the same way. Both edge weights and the number of edges were taken into account. For each HLA and peptide, the outcomes of each network were measured by four traditional centrality metrics: degree (the count of the number of other nodes that are adjacent [41]), betweenness (the frequency that a node falls on the shortest paths between pairwise nodes [41]), closeness (the inverse of the sum of the shortest paths from the node to all other nodes [41]) and eigenvector (the eigenvector of the largest eigenvalue of an adjacency matrix that represents the topology [42]). Originally, centrality metrics were used to identify the relative significance of individuals in social network analysis [33]. Here, the introduced metrics could be employed to describe the binding or immunogenicity propensities of the HLA-peptide pairs.

The acquired four centrality metrics for each model were scaled and then assigned to each HLA allele and peptide as new features that were used in the following deep learning model (see Deep learning network architecture part). Thus, for an HLA-peptide pair, eight new features were created (four for HLA and four for peptide).

#### The transformed network construction

To investigate the effectiveness of network metrics in the DeepNetBim model, we implemented two other transformed types of networks: shuffle networks and random networks for both the binding and immunogenic models. In the shuffle network, the network was reconstructed at the beginning by shuffling the edge weights at different levels (25%, 50%, 75% and 100% shuffle). The 25% shuffle model shuffled a quarter of the network edge weights, while the remaining three quarters of the network structure was unchanged. The 100% shuffle model shuffled all network edge weights. Such transformation only converted the connections of the network, while the overall values of weights were kept the same. Different from the shuffle model, the random model reassigned the network edge weights obeying a [0, 1] uniform distribution. We also acquired the corresponding four types of network metrics for further comparison with the original model. In the results section, we compared the output of the original model with that of the two transformed models to measure the effect of the network metrics.

### Sequence encoding

Peptide sequence data were encoded in terms of the BLOSUM50 scoring matrix [43], whose encoding scheme was widely used in previous studies [44–46]. As BLOSUM50 is a 20 * 20 substitution matrix, each amino acid is represented by its similarity to the other amino acids. Compared with the one-hot encoding strategy (i.e., encoded by 19 zeros and one), the BLOSUM matrix contains prior knowledge about subtle evolutionary and chemical relationships between the 20 amino acids [37, 47, 48]. In our work, peptides that contained amino acids “X”, “B”, and “U” were substituted by the character “Z”, which was not in the amino acid alphabet. Thus, each peptide was encoded to a 9*21 matrix, where each residue was represented by the corresponding row in the BLOSUM matrix. In our framework, the elements of the BLOSUM50 matrix were divided by a scaling factor of 10 to facilitate deep learning model training. In the DeepNetBim model, when predicting a newly added HLA-peptide pair in the network analysis, Euclidean distances between the new peptide and other encoded peptides in the training set were calculated accordingly. After that, the closest peptide network metrics in terms of Euclidean distances were chosen to represent metric features of the newly added peptides, and the median HLA network metrics were chosen to represent metric features of newly added HLA alleles.

### Deep learning network architecture

**Fig. 5:**
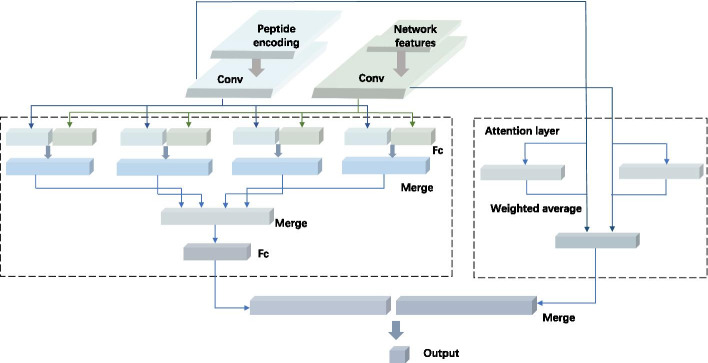
The architecture of the deep neural network in DeepNetBim. For an HLA-peptide pair, the encoded peptide (encoded to 9 × 21 matrix, coloured in blue) and network metrics (encoded to 1 × 8 vector, coloured in green) were fed into both convolutional and attention modules. The outputs of the two modules were then merged together by concatenating them to a single tensor

The DeepNetBim model passes encoded sequences and metrics features into combined modules of CNN and the attention module to make the final prediction. The attention-based deep neural network was implemented using the Keras library 2.2.4 (https://keras.io). Figure 5 shows the architecture of the deep learning network.

#### CNN module

In the DeepNetBim model, the peptide sequence was encoded into a 9 * 21 matrix, while the network metrics were encoded into a 1 * 8 matrix (four for HLA and four for the peptide). For the initial feature extraction, each of these two matrices was processed by a one-dimensional convolutional layer separately as input. For both peptide and network metric features, we used 256 convolutional filters of stride size 1 with 70% dropout to scan along the input sequence. The rectified linear unit (ReLU) was chosen as the activation function after the convolutional layer. Next, both peptide and network metric feature maps were flattened separately to obtain the long-form vectors. Then, each of the flattened vectors was concatenated with 4 identical copies before sending them to the corresponding fully connected layer. After that, two consecutive fully connected layers (with 64 and 4 output dimensions) were operated to generate the output of the convolutional layer.

#### Attention module

An attention layer was incorporated into our model to better improve the performance. The attention layer feeds each input feature to a shared neural network with a single hidden layer. It assigns different weights to the vectors and then computes their weighted average to facilitate the prediction. Briefly, it is expected that this will assign higher weights to the residues and positions that are more important in predicting peptide-MHC interactions [49]. The attention module is concatenated together with the output of the convolutional module and then forwarded into a fully connected layer with a sigmoid active function to obtain the final prediction.

The model was optimized and trained by minimizing the mean squared error loss function in the binding model and using the binary cross-entropy loss function in the immunogenic model with the Adam optimizer and RMSprop optimizer, respectively (minibatch size of 256). The optimal hyperparameters (including the number and size of convolutional filters, stride size for convolution, choice of activation function and size of fully connected layers) were determined through a grid search approach on the test set. A dropout layer was used to reduce overfitting by introducing noise in the CNN module, which led to a substantial increase in the overall performance.

### The combination of binding and immunogenic models

Considering both HLA binding and immunogenic prediction, we combined the two models by removing the predicted negative immunogenic HLA-peptide pairs (immunogenic category = 0). According to Eq. 3, if the predicted immunogenic category (IC) equals 1, we used the transformed predicted binding affinity (BA) as the combined score of our two models; otherwise, if the IC equals 0, the combined score corresponds to 0. Put simply, the combined model regards the use of the immunogenic model as an additional filter. In the results section, we surveyed the performance enhancement by the combined model.

3 $${\text{Combined score}} = \left\{ {\begin{array}{*{20}l} {{\text{BA}}} \hfill & {{\text{if }}\;{\text{IC}} = 1} \hfill \\ 0 \hfill & {{\text{if}}\;{\text{IC}} = 0} \hfill \\ \end{array} } \right.$$

### Fivefold cross-validation

Fivefold cross-validation was used to evaluate the model robustness. In fivefold cross validation, the dataset was randomly partitioned into five nonoverlapping subsets with each subset used as the test set, while the remaining subsets were used as the training set.

When there was an HLA or peptide in the testing set that never appeared in the training set, it was treated as newly added. In this way, the closest peptide network metrics and the median HLA network metrics were chosen to represent them (see the Sequence encoding section). Finally, when we applied our DeepNetBim method on the external datasets for prediction, all datasets were utilized for the training to achieve the best prediction performance.

## Supplementary Information

**Additional file 1: Figure S1.** The correlation of network metrics. The correlation among network metrics was higher amongst HLA attributes and amongst peptide attributes than between attributes of these two distinct groups. **Figure S2**. Density distribution of network metrics. Density distribution of HLA (a) and peptides (b) in the binding model and in the immunogenic model (c-d) where positives refer to binding/immunogenic and negatives refer to non-binding/non-immunogenic. **Figure S3.** Performance comparison between the original model and the submodels. The comparison between the original model and its submodels (each time dropping one of the network metrics from the full model) in the binding model (a) and the immunogenic model (b). **Figure S4.** The illustration of network construction. Each HLA and peptide was taken as a node. The network weight was assigned by the transformed affinity (in the binding model) or immunogenic binary category (in the immunogenic model).

**Additional file 2: Table S1.** Chi-square test on network metrics features on positive and negative datasets of binding and immunogenic model.

## Data Availability

The source codes supporting this work can be downloaded from https://github.com/Li-Lab-Proteomics/DeepNetBim or https://codeocean.com/capsule/4820141/tree/v1.

## Abbreviations

IEDB: Immune Epitope Database
HLA: Human leukocyte antigen
CNN: Convolutional neural network
PPV: Positive prediction value
MHC: Major histocompatibility complex
MS: Mass spectrometry
TAP: Transporter-associated with antigen processing
BA: Binding affinity
IC: Immunogenic category
HSV: Herpes simplex virus
SRCC: Spearman’s rank correlation coefficient
CV: Cross validation
