# Supplementary material for: DeepNetBim: deep learning model for predicting HLA-epitope interactions based on network analysis by harnessing binding and immunogenicity information
Source: BMC Bioinformatics. 2021 May 5;22:231. doi: 10.1186/s12859-021-04155-y (PMC8097772; doi:10.1186/s12859-021-04155-y)

HLA	Peptide	Transformed affinity
HLA1	Pep2	0.3
HLA2	Pep1	0.1
HLA2	Pep4	0.1
HLA3	Pep3	0.8
…	…	…

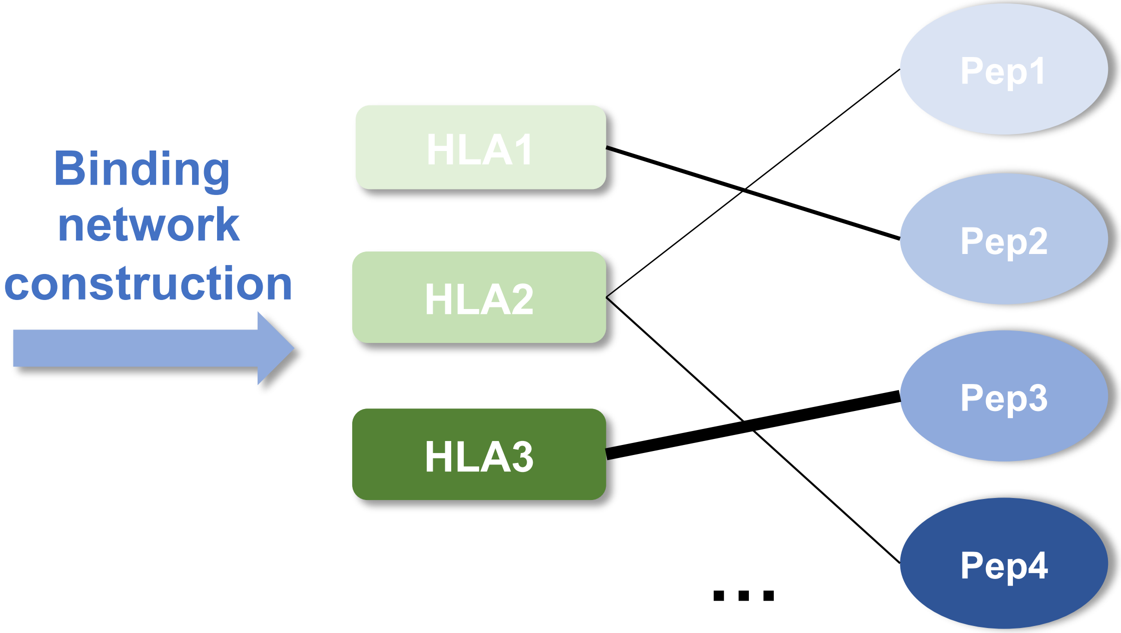

HLA	Peptide	Immunogenicity
HLA1	Pep2	1
HLA2	Pep1	0
HLA2	Pep4	1
HLA3	Pep3	1
…	…	…

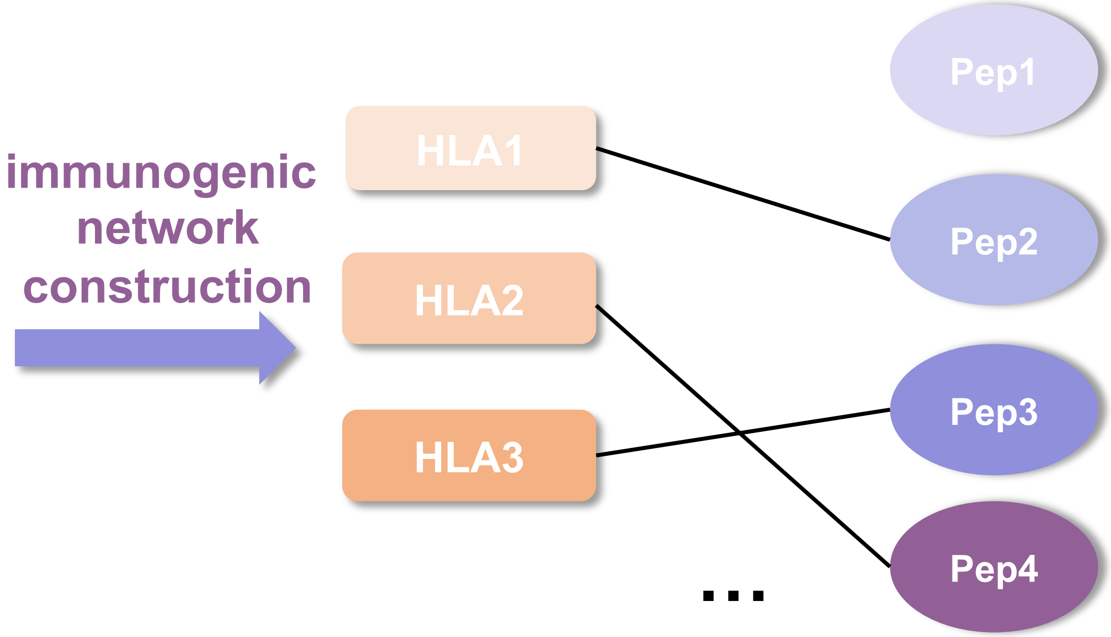

Supplement: Supplementary file 1 — Additional file 1: Figure S1. The correlation of network metrics. The correlation among network metrics was higher amongst HLA attributes and amongst peptide attributes than between attributes of these two distinct groups. Figure S2. Density distribution of network metrics. Density distribution of HLA (a) and peptides (b) in the binding model and in the immunogenic model (c-d) where positives refer to binding/immunogenic and negatives refer to non-binding/non-immunogenic. Figure S3. Performance comparison between the original model and the submodels. The comparison between the original model and its submodels (each time dropping one of the network metrics from the full model) in the binding model (a) and the immunogenic model (b). Figure S4. The illustration of network construction. Each HLA and peptide was taken as a node. The network weight was assigned by the transformed affinity (in the binding model) or immunogenic binary category (in the immunogenic model). [file 12859_2021_4155_MOESM1_ESM.zip › New folder/AdditionalFile1_SupplementaryFigure4.pdf]
